# TiO_2_ Nanotubes/Ag/MoS_2_ Meshy Photoelectrode with Excellent Photoelectrocatalytic Degradation Activity for Tetracycline Hydrochloride

**DOI:** 10.3390/nano8090666

**Published:** 2018-08-27

**Authors:** Tingting Li, Zhuhong Wang, Chaochao Liu, Chunmin Tang, Xinkai Wang, Gongsheng Ding, Yichun Ding, Lixia Yang

**Affiliations:** 1 College of Environmental and Chemical Engineering, Nanchang Hangkong University, Nanchang 330063, China; tingtingli1983@hotmail.com (T.L.); 13207180115@163.com (Z.W.); 15179207173@163.com (C.L.); 18296494686@163.com (C.T.); 18870922448@163.com (X.W.); dinggongsheng@126.com (G.D.); chempoem@126.com (Y.D.); 2 Key Laboratory of Jiangxi Province for Persisitent Pollutants Control and Resources Recyle, Nanchang Hangkong University, Nanchang 330063, China

**Keywords:** TiO_2_ nanotubes/Ag/MoS_2_, photoelectrode, photoelectrocatalytic degradation, tetracycline hydrochloride

## Abstract

A novel type of TiO_2_ nanotubes (NTs)/Ag/MoS_2_ meshy photoelectrode was fabricated with highly oriented TiO_2_ nanotube arrays grown from a Ti mesh supporting Ag nanoparticles and three-dimensional MoS_2_ nanosheets. In this structure, Ag nanoparticles act as bridges to connect MoS_2_ and TiO_2_ and pathways for electron transfer, ensuring the abundant production of active electrons, which are the source of •O_2_^−^. The TiO_2_ NTs/Ag/MoS_2_ mesh can be used as both photocatalyst and electrode, exhibiting enhanced photoelectrocatalytic efficiency in degrading tetracycline hydrochloride under visible light irradiation (λ ≥ 420 nm). Compared to unmodified TiO_2_ NTs, the improved photoelectrocatalytic activity of the TiO_2_ NTs/Ag/MoS_2_ arise from the formation of Z-scheme heterojunctions, which facilitate the efficient separation of photogenerated electron-hole pairs through the Schottky barriers at the interfaces of TiO_2_ NTs–Ag and Ag–MoS_2_.

## 1. Introduction

Over recent years, the presence and fate of antibiotics in the water environment has attracted considerable social attention due to threat they pose to human health and the safety of the ecological environment [1,2,3]. Tetracycline hydrochloride (TC·HCl) is the second most popular antibiotic, which has been widely used in clinical treatment and animal husbandry [4]. Backhaus et al. [5] found that TC·HCl was most toxic to aquatic microorganisms. TC·HCl cannot be biodegraded in traditional water treatment processes, and may hinder the removal of other organic pollutants [6]. Therefore, various methods have been employed to eliminate TC·HCl in the water environment, including advanced oxidation/reduction processes, adsorption, microbial degradation, and photocatalytic degradation [7,8,9,10]. Among them, semiconductor-based photocatalytic degradation is considered as an effective, energy-saving and sustainable technology to degrade TC·HCl under solar irradiation and ambient conditions [11,12].

A large number of semiconductor photocatalysts, such as modified TiO_2_ [6,13], BiWO_6_ [14], BiOI [15], Ag_3_PO_4_ [16], and Ag/AgIn_5_S_8_ [11] have been developed for photocatalytic degradation of TC·HCl. Although satisfactory removal efficiency of TC·HCl has been achieved with these photocatalysts, the addition of powdery chemicals results in difficult separation, poor reutilization, and extra pollution. In recent years, TiO_2_ nanotubes (NTs) fabricated by anodization of Ti foil have been widely studied due to their high photocatalytic activity, excellent chemical stability, and cyclic utilization. However, during photocatalysis, the reverse side of Ti foil cannot be excited by light, leading to deficient application of the catalyst. Moreover, TiO_2_ NTs grown vertically on Ti foil have the tendency to scatter incident light and cause the loss of photons. Furthermore, TiO_2_ NTs with lengths of more than 10 microns tend to peel away from Ti substrate due to the weak adhesion force on Ti foil, which is unfavorable for the photocatalysis. As a result, Ti mesh was selected in this study as an ideal alternative to solve these disadvantages. Unlike planar Ti foil where nanotubes are grown vertically in two-dimensional (2D) arrays, the TiO_2_ NTs/Ti mesh exhibit three-dimensional (3D) arrays grown from a grid of fine titanium wires [17]. Therefore, the loss of photons attributed to scattering effects in the liquid can be remarkably minimized because the nanotubes grown surrounding the Ti wires can absorb reflected light from different directions [18]. Compared with Ti foils, TiO_2_ NTs formed over Ti meshes can harvest more light. Moreover, anodic TiO_2_ NTs/Ti mesh can be used as both photocatalyst and electrode. Under light irradiation, TiO_2_ NTs/Ti mesh can serve as photocatalyst to generate photogenerated electrons (e^−^)-holes (h^+^) pairs, which are prone to recombine rather than participate in redox reaction of target pollutants [19]. Since TiO_2_ NTs/Ti mesh can also work as anode, the photogenerated electrons produced in TiO_2_ NTs can rapidly transfer to the positive pole of external circuit under the assistance of external electric field, which effectively restrains the recombination of e^−^-h^+^ pairs. As a result, the quantum efficiency of photoinduced carriers is enhanced, and the final photoelectrocatalytic efficiency is greatly improved [20].

However, the application of TiO_2_ NTs in photocatalysis is restricted by its low visible light utilization due to the wide band gap of TiO_2_ (3.2 eV for anatase and 3.0 eV for rutile). Various attempts have been made to improve the absorption efficiency of visible light, such as transition metal cations doping [21], nonmetal anions doping [22], surface modification with noble metal [23], and semiconductor heterojunctions [24]. In terms of the modified noble metal, silver nanoparticles (Ag NPs) with localized surface plasmon resonance (LSPR) effect is one of the most suitable candidates for assistant photocatalysts due to its low cost and high activity [25]. Furthermore, MoS_2_ is a graphene-like layered transition metal dichalcogenides with an appropriate band gap of 1.17 eV, which is small enough to narrow the band gap of TiO_2_ to extend its wavelength response range to the visible region [26]. As a result, the coupling of a wide band gap semiconductor (TiO_2_ NTs) with two suitable narrow band gap ones (Ag and MoS_2_) leads to the formation of TiO_2_ NTs/Ag/MoS_2_ heterojunctions, which will be highly effective in improving the utilization of solar light, promoting interfacial charge transfer, and thus enhancing photoelecrocatalytic activity for removing TC·HCl.

In this study, a conductive Ti mesh bearing highly ordered and oriented TiO_2_ NTs was employed as solid substrates for the assembly of Ag nanoparticles and MoS_2_ nanosheets through photochemical reduction and hydrothermal methods. The Ag nanoparticles and MoS_2_ nanosheets were uniformly distributed on the top surface of TiO_2_ NTs rather than filling in the nanotubes, thus allowing these tubular channels open to the environment, which was beneficial for effective separation of photogenerated e^−^-h^+^ pairs. Under visible irradiation and applied voltage, the resulting TiO_2_ NTs/Ag/MoS_2_ mesh worked as photoelectrode and exhibited superior photoelectrocatalytic activity for the degradation of TC·HCl. In addition, a possible photoelectrcatalytic degradation mechanism was proposed by identifying reactive species involved and electron spin resonance (ESR) spectra.

## 2. Experimental Section

### 2.1. Preparation of TiO_2_ NTs, TiO_2_ NTs/Ag, TiO_2_ NTs/MoS_2_ and TiO_2_ NTs/Ag/MoS_2_ Meshy Photoelectrode

TiO_2_ NTs meshy photoelectrode was prepared via electrochemical anodic oxidation. Titanium meshes (99.8%) with the size of 1 cm × 4 cm and a thickness of 0.1 mm were ultrasonically cleaned with acetone, ethanol, and deionized (DI) water. A two-electrode electrochemical cell was employed for the anodization using Ti mesh as the working electrode and platinum foil as the counter electrode. The Ti mesh was anodized under 30 V for 8 h in the electrolyte of *N*,*N*-dimethyl sulfoxide containing 2 wt % HF. After anodization, the samples were washed with DI water and then annealed at 500 °C in air atmosphere for 3 h to convert amorphous TiO_2_ to anatase phase.

TiO_2_ NTs/Ag meshy photoelectrode was fabricated by photochemical reduction method. In a typical process, the TiO_2_ NTs/Ti mesh substrate was soaked in 0.006 M AgNO_3_ aqueous solution under ultrasonication for 30 min and then dried in air. The mesh was then immersed in 0.1 M methanol solution and irradiated under the 300 W Xe lamp for 30 min to reduce adsorbed Ag^+^ to Ag^0^.

TiO_2_ NTs/MoS_2_ meshy photoelectrode was synthesized by a facile hydrothermal reaction. Typically, 10 mg of sodium molybdate (Na_2_MoO_4_·2H_2_O) and 30 mg of thioacetamide (C_2_H_5_NS) were dissolved in 20 mL DI water to form a transparent solution. The resulting solution was transferred into a 50 mL Telfon-lined stainless steel autoclave, and the TiO_2_ NTs/Ti mesh substrate was vertically immersed in the solution. Subsequently, the autoclave was heated in an oven at 200 °C for 24 h. After cooling down to room temperature, the product was washed with DI water and dried at 80 °C for 12 h. The as-prepared sample was annealed at 450 °C in N_2_ atmosphere for 2 h to obtain the highly crystalline MoS_2_.

TiO_2_ NTs/Ag/MoS_2_ meshy photoelectrode was prepared through a similar hydrothermal process and calcination using the TiO_2_ NTs/Ag mesh as substrate.

### 2.2. Characterization

The morphologies and microstructures of the products were characterized by field emission scanning electron microscopy (FESEM, S-4800, Hitachi Tokyo, Japan) and transmission electron microscopy (TEM, JEOL3010, JEOL Ltd., Akishima, Tokyo, Japan). The crystalline phase of the samples was determined by X-ray powders diffraction (XRD) using a Bruker D8 X-ray diffractometer (Bruker, Billerica, MA, USA) with CuK_α_ (λ = 0.15418 nm) in the 2θ range of 10° to 80°. X-ray photoelectron spectroscopy (XPS, Thermo ESCALAB250, Thermo Scientific, Waltham, MA, USA) was employed to examine the surface properties and chemical composition of the samples. The Brunauer–Emmett–Teller (BET) surface area was measured using a TriStar II 3020 Surface Area and Porosity Analyzer (Micromeritics Instrument Corporation, Norcross, GA, USA). The UV-vis diffuse reflection spectra (DRS) were collected on a U-3900H spectrophotometer (Hitachi, Tokyo, Japan) equipped with integrating sphere accessory, using BaSO_4_ as reference. The photoluminescence (PL) spectra were recorded with fluorescence spectrophotometer (Hitachi, F-7000, Tokyo, Japan) at an excitation wavelength of 380 nm.

### 2.3. Photoelectrochemical Measurements

The photocurrent measurements were carried out using an electrochemical workstation (CHI660C, ShanghaiChenhua Co., Ltd., Shanghai, China) with a standard three-electrode configuration with the as-prepared meshy photoelectrode as working electrode, a KCl-saturated calomel electrode (SCE) as reference electrode, and a platinum foil as counter electrode. A 300 W Xe lamp (PLS-SXE300UV, Beijing Trusttech Co. Ltd., Beijing, China) with a 420 nm cut-off filter was employed as the source of visible light (λ ≥ 420 nm). The time-dependent photocurrent response was measured in an electrolyte containing 0.5 mol/L Na_2_SO_4_ at a fixed bias of 0 V versus SCE under chopped visible-light irradiation (light/dark cycles of 50 s).

Electrochemical impedance spectroscopy (EIS) measurements were recorded using the same three-electrode configuration at an AC voltage magnitude of 0 mV with the frequency range of 10^5^–1 Hz. The supporting electrolyte was a solution containing 0.5 mol/L Na_2_SO_4_, 2.5 mmol/L potassium hexacyanoferrate (III) (K_3_[Fe(CN)_6_]), and 2.5 mmol/L potassium ferrocyanide (K_4_[Fe(CN)_6_]).

### 2.4. Photoelectrocatalytic Degradation Experiments

Photoelectrocatalytic removal of TC·HCl with the meshy photoelecrodes was performed in a photoelectrochemical reactor. The as-prepared meshy photoelectrodes with an effective geometrical area of 4.0 cm^2^ was used as working electrode, a platinum foil as counter electrode, and 80 mL of 10 mg/L TC·HCl solution as target pollutant. A DC power (Array 3646A, Taiwan, China) supplied electricity with a bias voltage of 0.1 V. Prior to irradiation, the solution was magnetically stirred in the dark for 60 min to establish the adsorption–desorption equilibrium. Then, the samples were evaluated by degradation of TC·HCl solution under visible irradiation (λ ≥ 420 nm). In every 30 min time interval, 3 mL of the solution was removed, and the concentration changes of TC·HCl were analyzed by checking the absorbance at the characteristic adsorption peak of 358 nm.

## 3. Results and Discussion

### 3.1. Morphological and Structural Characterization

**Figure 1:**
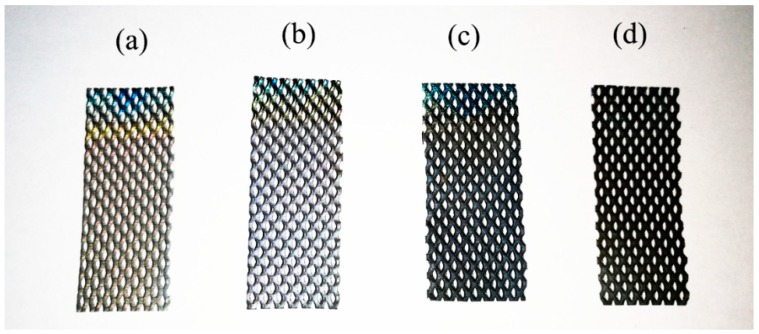
Digital pictures of (**a**) TiO_2_ nanotubes (NTs); (**b**) TiO_2_ NTs/Ag; (**c**) TiO_2_ NTs/MoS_2_; and (**d**) TiO_2_ NTs/Ag/MoS_2_ mesh.

The photographs of four meshy photoelectrodes based on Ti mesh substrates are shown in Figure 1. The TiO_2_ NTs meshy photoelectrode (Figure 1a) exhibited a silver color; after being deposited with Ag nanoparticles, it turned into light blue with a metallic luster (Figure 1b). After being decorated by MoS_2_ nanosheets, the resulting TiO_2_ NTs/Ag/MoS_2_ meshes appeared black (Figure 1c,d), which is beneficial for improving the capability in harvesting light.

**Figure 2:**
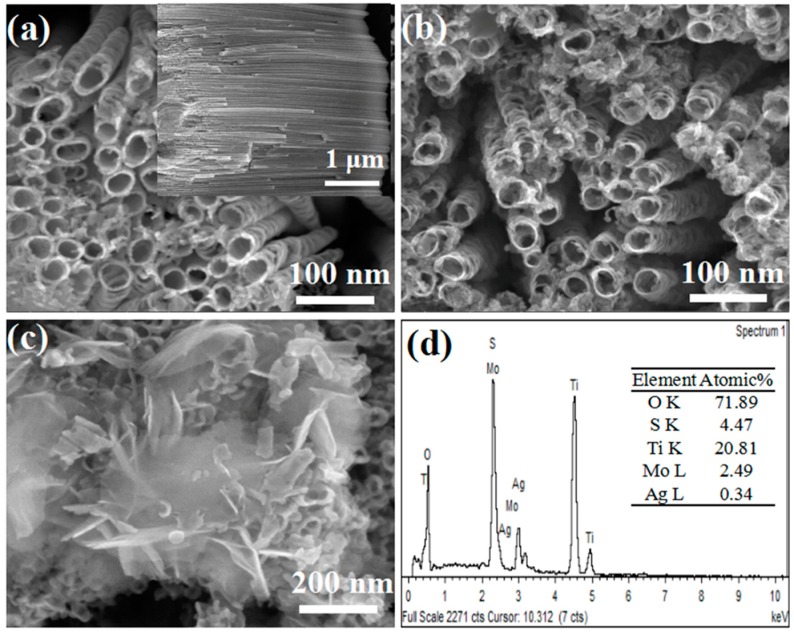
SEM images of (**a**) TiO_2_ NTs; (**b**) TiO_2_ NTs/Ag; and (**c**) TiO_2_ NTs/Ag/MoS_2_; (**d**) EDS analysis of TiO_2_ NTs/Ag/MoS_2_.

The morphologies of as-prepared meshy photoelectrodes were characterized by SEM. Figure 2a exhibits that the pure TiO_2_ NTs had vertically oriented tubular structure with a bamboo-like appearance. The nanotubes were 40–50 nm in diameter and 4–5 μm in length. The TiO_2_ NTs/Ag depicted in Figure 2b shows that Ag nanoparticles were evenly distributed on top surface and the tube walls of the TiO_2_ NTs (Figure 2b), which acted as nucleation centers for the in situ growth of MoS_2_ nanocrystalline. As shown in Figure 2c, MoS_2_ nanosheets were grown over the TiO_2_ NTs/Ag well-constructed morphology, which interconnected with each other closely to form three-dimensional networks. Here, the anatase TiO_2_ NTs after thermal annealing treatment was highly conductive [27]. The TiO_2_ NTs could serve as backbone for the in situ growth of MoS_2_ nanosheets with random elastic defomations and distortion edges, which constructed a stable hybrid configuration with a lot of exposed active sites [28]. The EDS spectrum (Figure 2d) of the TiO_2_ NTs/Ag/MoS_2_ confirmed the presence of O, S, Ti, Mo, and Ag elements. Quantitative analysis of EDS gave the deposited composite a possible composition of MoS_2_.

**Figure 3:**
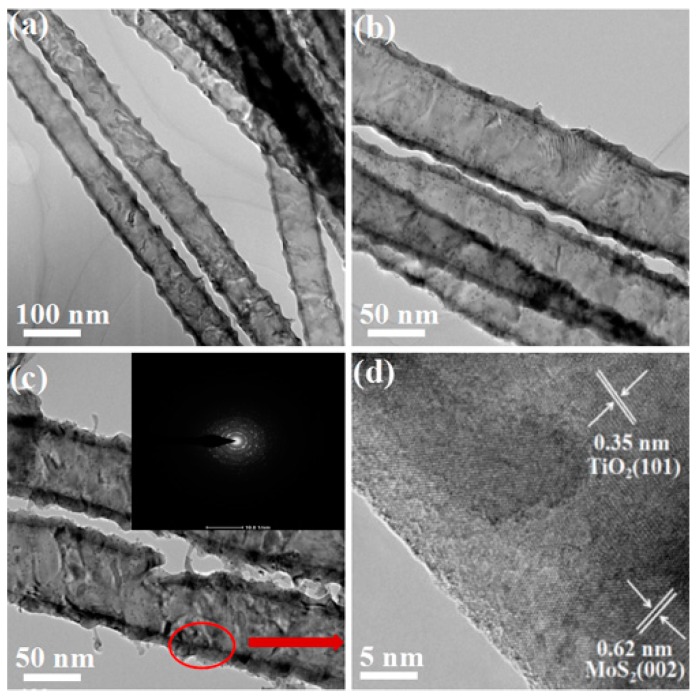
TEM images of (**a**) TiO_2_ NTs; (**b**) TiO_2_ NTs/Ag; and (**c**) TiO_2_ NTs/Ag/MoS_2_ (inset: SAED pattern); (**d**) HRTEM image of TiO_2_ NTs/Ag/MoS_2_.

The microstructures of the resulting TiO_2_ NTs, TiO_2_ NTs/Ag and TiO_2_ NTs/Ag/MoS_2_ were further identified by TEM and HRTEM (JEOL Ltd., Akishima, Tokyo, Japan) observations. As depicted in Figure 3a, the unmodified TiO_2_ NTs were highly ordered and transparent with diameter of 50 nm and wall thickness of 10 nm, in agreement with the SEM observations in Figure 2a. TEM image in Figure 3b confirmed that Ag nanoparticles were uniformly and densely loaded on both the internal and external walls of the TiO_2_ NTs without blocking at the top openings (Figure 3b). The uniform distribution of Ag nanoparticles enabled the fast transfer of the photogenerated electrons, which contributed to reduce the combination probability of photogenerated e^−^-h^+^ pairs on the TiO_2_ NTs and enhance the quantum efficiency. The TEM image in Figure 3c exhibits that the decoration of Ag and MoS_2_ had no significant influence on the tubular structure of TiO_2_ NTs, indicating a good combination between the MoS_2_ and TiO_2_ crystals. Due to the inherent resistance of TiO_2_, the deposition of foreign species appeared preferentially on the outside rather than the inside of nanotubes [26]. Few-layer MoS_2_ nanosheets surrounded the tube openings and the intertubular gaps between the TiO_2_ NTs, leaving a large portion of tube surface accessible to the outer environment; this facilitated the exposure of active sites for absorbing and decomposing target molecules. The selected area electron diffraction (SAED) pattern (inset in Figure 3c) suggested that the TiO_2_ NTs that combined the Ag nanoparticles and MoS_2_ nanosheets was an ideal hybrid. The typical HRTEM image of the TiO_2_ NTs/Ag/MoS_2_ is shown in Figure 3d. The well-bounded lattice spacing of 0.35 nm corresponded to the (101) plane of anatase structure, which was the major exposed lattice plane of anatase TiO_2_ [27]. Besides, the (002) lattice plane of hexagonal 2H-MoS_2_ with a lattice spacing of 0.62 nm was also observed. To further confirm the distribution of Ti, O, Mo, S, and Ag elements on the TiO_2_ NTs/Ag/MoS_2_, element mappings are supplied in Figure S1. The Ti and O mappings showed obvious bright edge and dark inside, demonstrating a distinct tubular structure. The color signals of Mo, S, and Ag mappings were evident, suggesting that MoS_2_ nanosheet and Ag nanoparticles were homogeneously deposited on TiO_2_ NTs.

**Figure 4:**
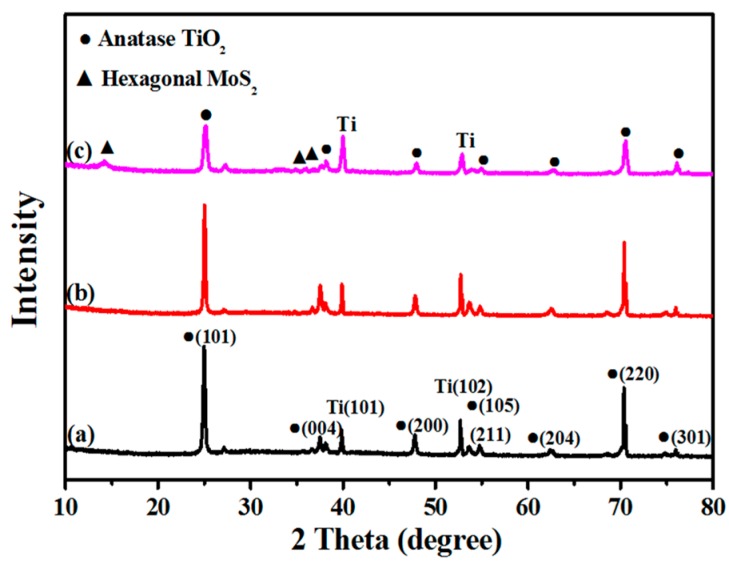
XRD patterns of (a) TiO_2_ NTs; (b) TiO_2_ NTs/Ag; and (c) TiO_2_ NTs/Ag/MoS_2_.

Figure 4 depicts the XRD patterns of the meshy photoelectrodes: the TiO_2_ NTs, TiO_2_ NTs/Ag, and TiO_2_ NTs/Ag/MoS_2_ composites. The diffraction peaks of pure TiO_2_ NTs (Figure 1a) were indexed to (101), (004), (200), (105), (211), (204), (220), and (301) crystal planes of anatase phase (JCPDS No. 21–1272). A major peak located at 25.3° can be ascribed to (101) plane, which is the most thermodynamically stable crystal face of anatase TiO_2_. However, the characteristic peaks assigned to Ag^0^ (JCPDS No. 65–2871) were not observed in the diffraction peaks of the TiO_2_ NTs/Ag (Figure 1b), probably due to its low content, poor crystallinity, and high dispersity. As for the TiO_2_ NTs/Ag/MoS_2_ (Figure 1c), the characteristic peaks emerged at 14.4, 33.5, and 35.8 eV, which could be assigned to the (002), (101), (102) lattice plane of the hexagonal phase 2H-MoS_2_ (JCPDS card No. 37–1492) [29]. As reported, the growth of MoS_2_ along the (002) plane might be inhibited by the pure TiO_2_ NTs during the hydrothermal process, so the assembly of ultrathin MoS_2_ layers results in the formation of nanosheets rather than nanoflowers [27,30].

**Figure 5:**
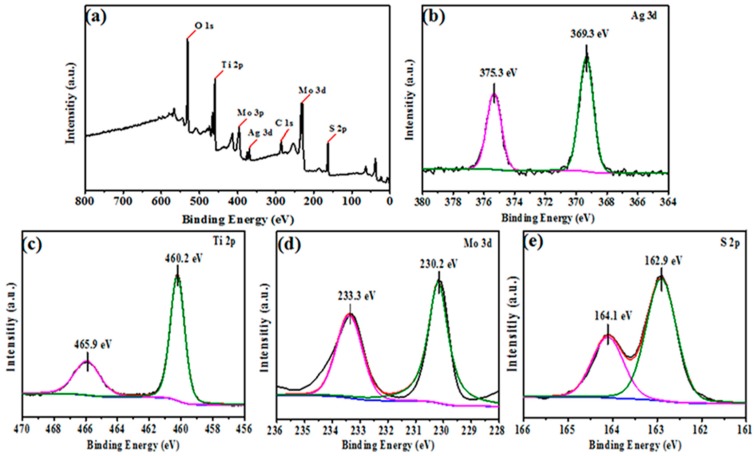
X-ray photoelectron spectroscopy (XPS) spectra of TiO_2_ NTs/Ag/MoS_2_: (**a**) survey, (**b**) Ag 3d, (**c**) Ti 2p, (**d**) Mo 3d, and (**e**) S 2p.

To further elucidate the surface elemental composition and chemical states present in the TiO_2_ NTs/Ag/MoS_2_, surface analysis was performed using XPS technique. The full survey spectrum, shown in Figure 5a, revealed that the composite was composed of O, Ti, Mo, Ag, and S elements. As shown in typical high-resolution XPS spectrum of Ag 3d (Figure 5b), the Ag 3d 3/2, and Ag 3d 5/2 peaks were identified at 369.3 eV and 375.3 eV, and the splitting of the 3d doublet was 6.0 eV. This binding energy indicated that Ag mainly existed in the Ag^0^ state on the TiO_2_ NTs [25]. The Ti 2p peaks located at 460.2 and 465.9 eV (Figure 5c) with a spin-orbital doublet splitting (Ti 2p3/2-Ti 2p1/2) of 5.7 eV, which indicated an oxidation state of Ti^4+^ in TiO_2_ [31]. In Figure 5d, the Mo 3d spectrum shows two primary peaks at 230.2 and 233.3 eV, which can be assigned to the doublet Mo 3d 5/2 and Mo 3d 3/2 of Mo 4p [32]. The S 2p spectrum showed two primary peaks at 162.9 and 164.1 eV (Figure 5e), which can be attributed to the S 2p3/2 and S 2p1/2 orbitals of divalent sulfide ions (S^2−^) of MoS_2_ [33]. For the TiO_2_ NTs/Ag/MoS_2_ composites, the fitted peaks shifted to negative higher energies of ≈1.5 eV, suggesting the electronic interaction among MoS_2_, Ag, and TiO_2_ [23,27]. The XPS results further confirmed the MoS_2_ and Ag were successfully incorporated into the TiO_2_ NTs, which are in good agreement with the XRD results.

**Figure 6:**
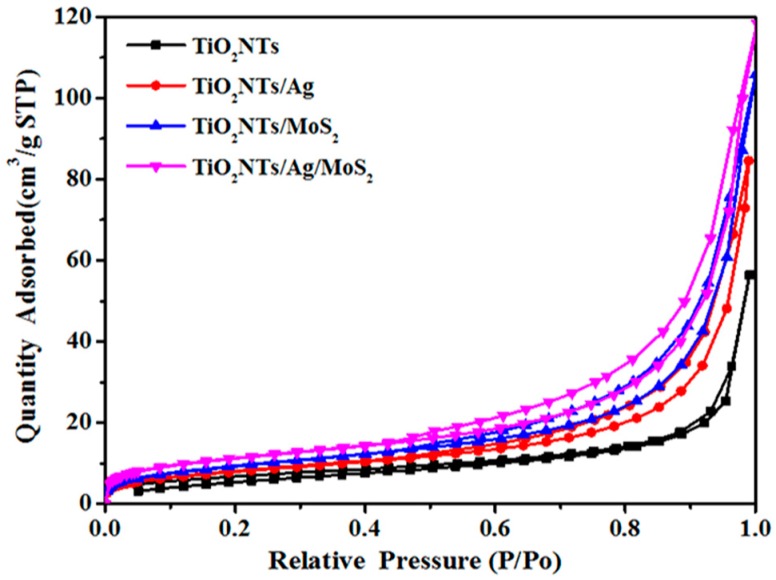
Nitrogen absorption–desorption isotherms of TiO_2_ NTs, TiO_2_ NTs/Ag, TiO_2_ NTs/MoS_2_, and TiO_2_ NTs/Ag/MoS_2_.

The Brunauer–Emmett–Teller specific surface area of the as-synthesized meshy photoelectrodes were measured by the nitrogen adsorption and desorption isotherms. As exhibited in Figure 6, the isotherms of the samples could be classified to a type-IV isotherm (based on IUPAC recommendation) with a H3 hysteresis loop at P/P_0_ > 0.6 (BDDT classification). In comparison to the BET surface area of the TiO_2_ NTs (21.6 m^2^/g), the BET surface area of the TiO_2_ NTs/Ag, TiO_2_ NTs/MoS_2_, and TiO_2_ NTs/Ag/MoS_2_ increased gradually (29.1 m^2^/g, 34.5 m^2^/g and 40.2 m^2^/g). The larger BET surface area and porous framework of the TiO_2_ NTs/Ag/MoS_2_ can provide more active sites and improve the flow rate of both target molecules and the entry and multireflections of visible light, which can be favorable to the enhancement of photocatalytic activity.

### 3.2. Photoelectrochemical Measurements

**Figure 7:**
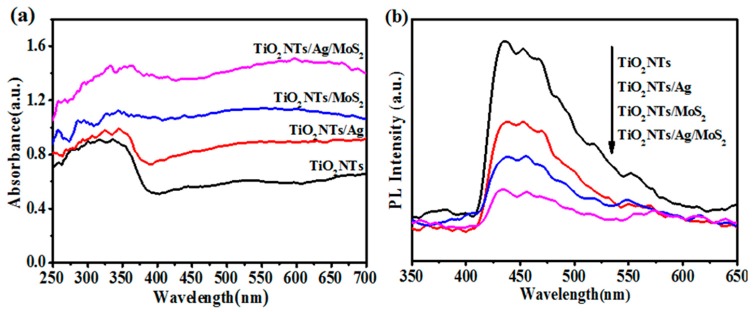
(**a**) UV-vis diffuse reflectance spectra; (**b**) PL spectra of TiO_2_ NTs, TiO_2_ NTs/Ag, TiO_2_ NTs/MoS_2_, and TiO_2_ NTs/Ag/MoS_2_.

The UV-vis diffuse reflectance spectra of the as-prepared meshy photoelectrodes are depicted in Figure 7a. The pure TiO_2_ NTs only responded to the ultraviolet light. After being modified by Ag or MoS_2_, not only was the UV-vis light adsorption of TiO_2_ NTs obviously enhanced but a red shift of the absorption edge to the visible region was also observed. The TiO_2_ NTs/Ag/MoS_2_ displayed the strongest optical absorption intensity in the light range of 400–700 nm. This is because the black MoS_2_ is beneficial for improving the response to the visible light. Furthermore, the enhanced visible-light absorption is also caused by the metal-like local surface plasma resonance effect, which arises from collective oscillations of excess electrons on the edge of MoS_2_ [34].

The photoluminescence (PL) spectrum was employed to evaluate the recombination rate of the photogenerated e^−^-h^+^ pairs. As shown in Figure 7b, the pure TiO_2_ NTs exhibited a distinct emission peak centered at 450 nm, which can be ascribed to the electrons trapped at shallow level defects [35]. The ternary TiO_2_ NTs/Ag/MoS_2_ exhibited the lowest PL intensity, indicating that the electron-hole recombination of self-trapped excitation in TiO_2_ NTs was greatly suppressed by the introduction of Ag and MoS_2_.

**Figure 8:**
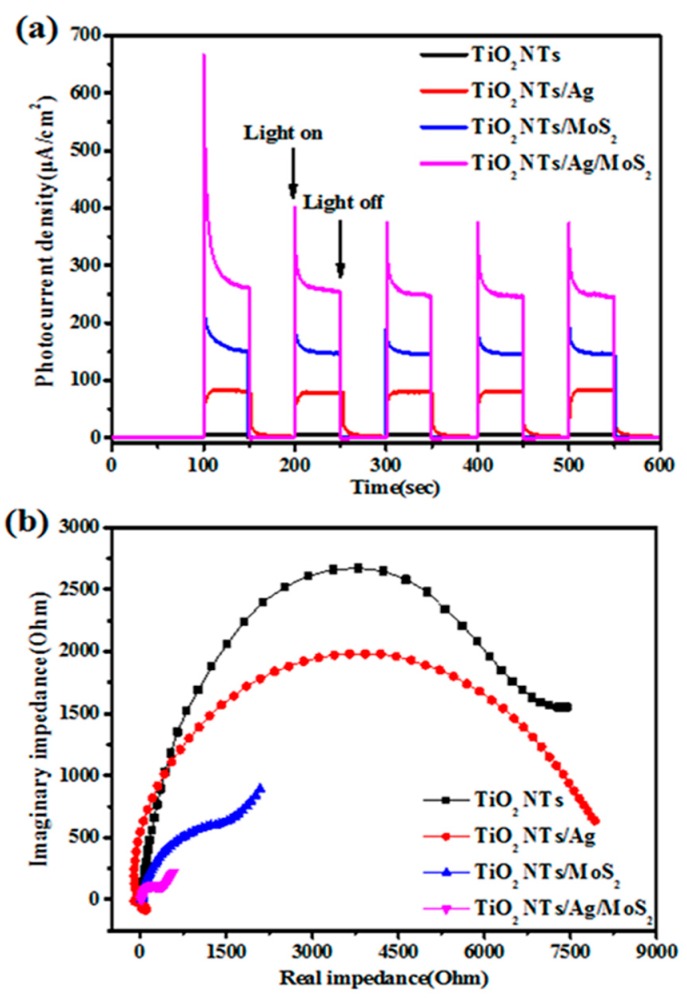
(**a**) Photocurrent responses; (**b**) electrochemical impedance spectroscopy (EIS) Nyquist plots of TiO_2_ NTs, TiO_2_ NTs/Ag, TiO_2_ NTs/MoS_2_, and TiO_2_ NTs/Ag/MoS_2_.

To further verify the separation rate of photogenerated charge carriers, the photocurrent responses of the four meshy photoelectrodes were determined. As shown in Figure 8a, the photocurrent densities promptly increased or decreased when light was turned on or off. The lowest photocurrent density was recorded over the TiO_2_ NTs due to fast recombination of e^−^-h^+^ pairs. The TiO_2_ NTs/Ag/MoS_2_ exhibited the highest photocurrent density of 0.25 mA cm^−^^2^, which can be ascribed to the efficient separation of the photogenerated e^−^-h^+^ pairs on the interfaces of TiO_2_ NTs–Ag and Ag–MoS_2_.

Electrochemical impedance spectra (EIS) was performed to study the electrochemical properties of the as-prepared meshy photoelectrodes. As depicted in Figure 8b, the arc radius on the Nyquist plot of the TiO_2_ NTs/Ag/MoS_2_ was the smallest among the four meshy photoelectrodes under visible light irradiation, indicating that the introduction of Ag and MoS_2_ effectively decreased the resistance of the TiO_2_ NTs and consequently accelerated the electron transfer velocity on the meshy photoelectrode.

### 3.3. Photoelectrocatalytic Activity of the Meshy Photoelectrodes

**Figure 9:**
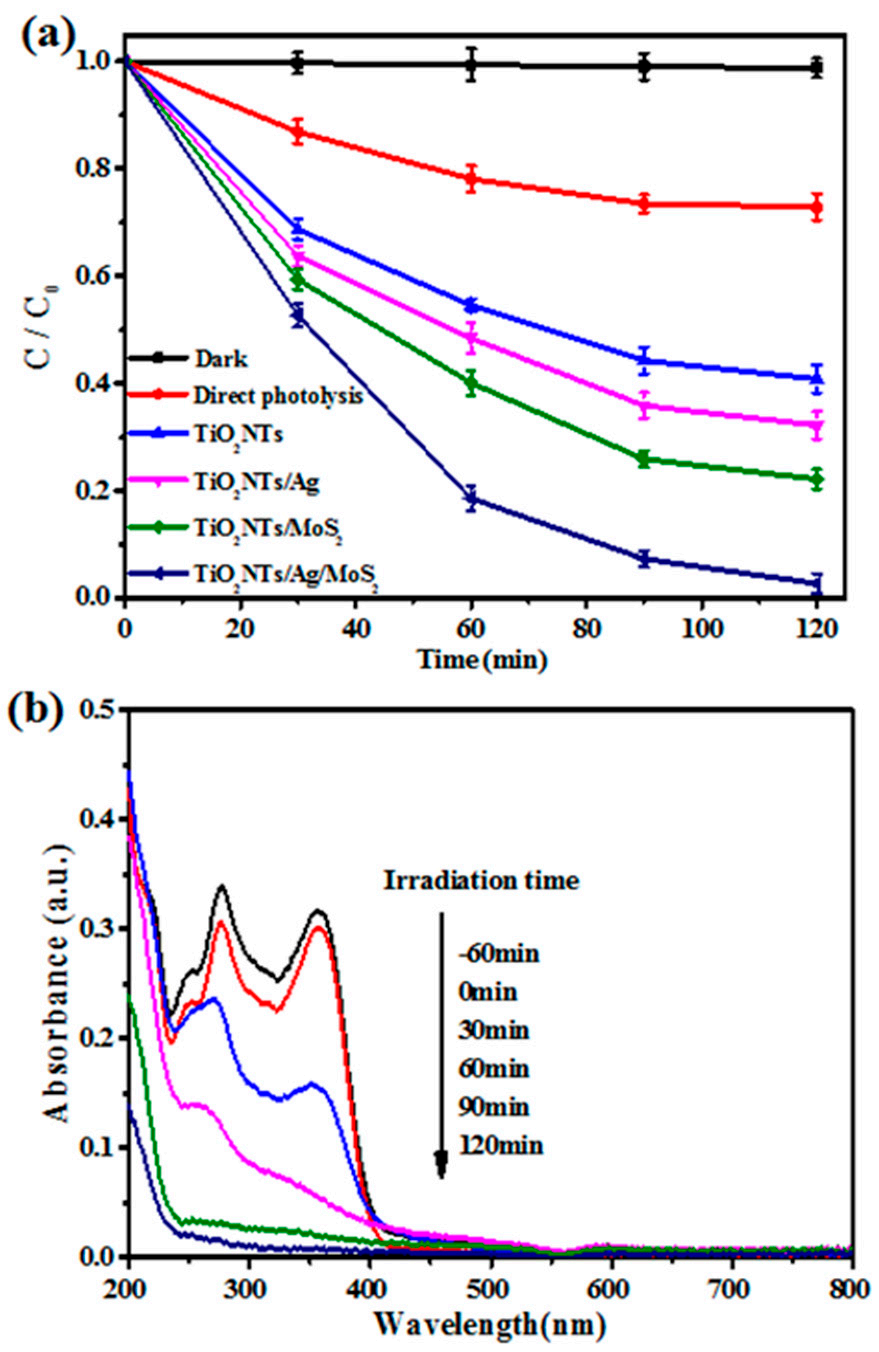
(**a**) The photoelectrocatalytic (PEC) efficiencies of TC·HCl under different conditions; (**b**) UV-vis absorption spectra for the degradation of TC·HCl on the TiO_2_ NTs/Ag/MoS_2_.

The photoelectrocatalytic (PEC) activities of the as-prepared samples were evaluated by the degradation of TC·HCl under visible light irradiation. As shown in Figure 9a, the degradation of TC·HCl was negligible in dark and direct photolysis. After irradiation for 120 min, 60.1%, 68.3%, 78.1%, and 97.2% of TC·HCl were removed by the TiO_2_ NTs, TiO_2_ NTs/Ag, TiO_2_ NTs/MoS_2_, and TiO_2_ NTs/Ag/MoS_2_ meshy photoelectrodes, respectively. Figure 9b exhibits the UV-vis spectra of TC·HCl with respect to irradation durations in the presence of the TiO_2_ NTs/Ag/MoS_2_ meshy photoelectrode. The intensity of characteristic peaks of TC·HCl diminished gradually as irradiation time increased and nearly disappeared within 120 min. These results demonstrate that TC·HCl can be effectively degradated by TiO_2_ NTs/Ag/MoS_2_ under visible light.

The PEC activity of the TiO_2_ NTs/Ag/MoS_2_ meshy photoelectrode was evaluated by applying different voltage of 0.05 V, 0.1 V, 0.2 V, 0.3V, and 0.5 V. As depicted in Figure S2a, the TiO_2_ NTs/Ag/MoS_2_ showed the highest degradation efficiency with the applied voltage of 0.1 V. Figure S2b shows the removal efficiency of TC·HCl under different degradation processes, including electrochemical catalysis (EC) at 0.1 V bias potential, photocatalysis (PC), and PEC. The PEC removal efficiency of TC·HCl was much higher than that in the PC and EC processes. With assistance of both light excitation and applied bias potential, the electron density on the TiO_2_ NTs/Ag/MoS_2_ was much thicker than those in EC and PC processes, which ensured the effective degradation of TC·HCl and achieved the highest catalytic efficiency.

### 3.4. Study on Catalyst Stability and Photoelectrocatalytic Mechanism

**Figure 10:**
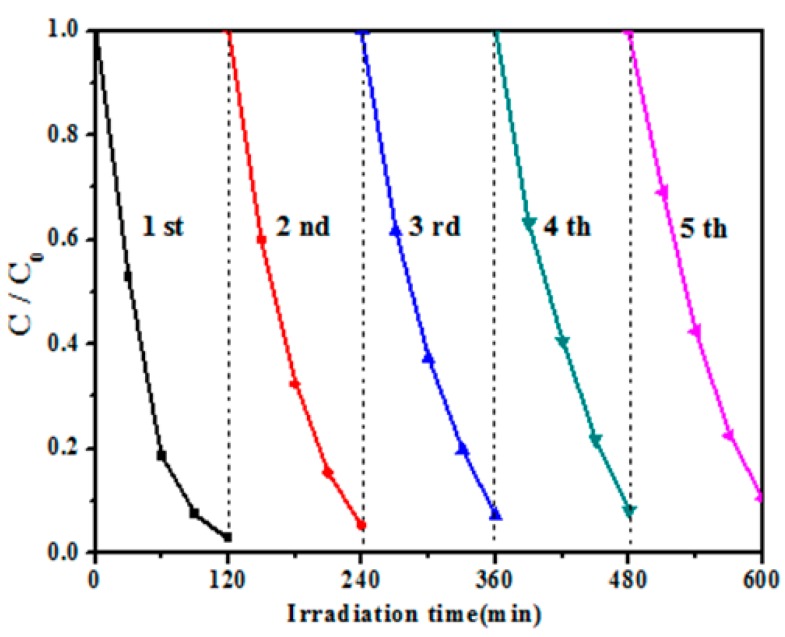
Cycling runs in the PEC degradation of TC·HCl over the TiO_2_ NTs/Ag/MoS_2_.

The results of the recycle experiments of the TiO_2_ NTs/Ag/MoS_2_ are exhibited in Figure 10. There was a slight loss of 8% in PEC activity after five runs for the degradation of TC·HCl. Additionally, the XRD pattern of the fresh and used TiO_2_ NTs/Ag/MoS_2_ during the PEC process in Figure S3 revealed that the crystal phase had not changed significantly. This suggests that the TiO_2_ NTs/Ag/MoS_2_ meshy photoelectrode possesses excellent stability and reliability for PEC degradation of TC·HCl.

**Figure 11:**
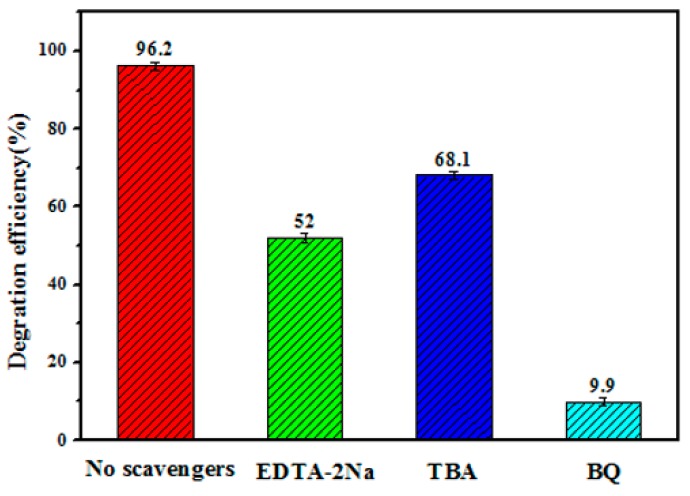
The PEC efficiencies of TC·HCl with different scavengers over the TiO_2_ NTs/Ag/MoS_2_: no scavenger; with 1 mM ethylenediamintetraacetic acid disodium (EDTA-2Na); with 1 mM tert-butyl alcohol (TBA); and with 1 mM 1,4-benzoquinone (BQ).

In order to explore the predominant active species in the PEC process, 1,4-benzoquinone (BQ), ethylenediamintetraacetic acid disodium (EDTA-2Na) and tert-butyl alcohol (TBA) were applied as the scavengers of superoxide radical (•O_2_^−^) [36], hole (h^+^) [37] and hydroxyl radical (•OH) [38], respectively. As illustrated in Figure 11, the addition of BQ exhibited strong inhibition on the PEC activity of the TiO_2_ NTs/Ag/MoS_2_, suggesting that •O_2_^−^ radicals played an important role in the degradation of TC·HCl. When EDTA-2Na and TBA were added, the degradation efficiency of TC·HCl decreased from 96.2% to 52.0% and 68.1%, respectively, indicating that h^+^ and •OH radicals are minor reactive species.

The spin-trapping ESR technique (with DMPO) was employed to confirm •OH and •O_2_^−^ radicals generated over the TiO_2_ NTs/Ag/MoS_2_ (Figure S4). Under visible light irradiation, the signals of DMPO-•OH were demonstrated by quartet lines with peak intensity of 1:2:2:1. In addition, four characteristic peaks of DMPO-•O_2_^−^ were observed under visible light irradiation, but no such signals were detected in the dark. The results indicate that the TiO_2_ NTs/Ag/MoS_2_ meshy photoelectrode can be excited by visible light to produce e^−^-h^+^ pairs. Moreover, the charge separation is efficient enough to allow photogenerated electrons or holes to react with adsorbed O_2_ or H_2_O molecules to generate active radicals such as •OH and •O_2_^−^. 

**Scheme 1:**
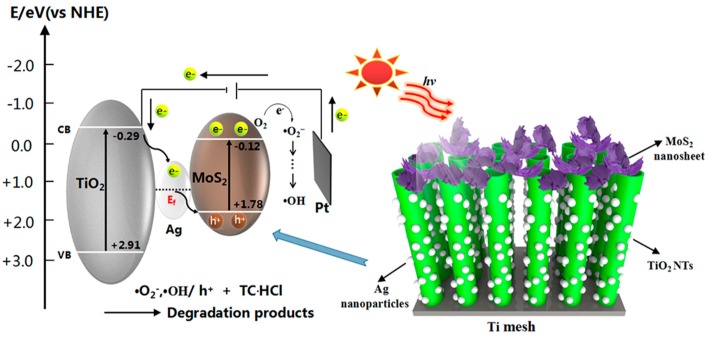
Schematic illustration of proposed mechanism of the photogenerated electrons and holes transfer in the TiO_2_ NTs/Ag/MoS_2_ interface.

As a recent paper depicted [39], there are two mechanisms related to the charge transfer path across the interface in TiO_2_-based heterojunctions, including a double-charge transfer and a direct Z-scheme mechanism. The double-charge transfer mechanism favors charge separation at the expense of a decrease in the potential energy of photogenerated electrons and holes. The Z-scheme mechanism supports the electrons transfer across the interface, which could provide a way for preserving photostability while maintaining a high reduction potential. In this work, on the basis of above experimental data and theoretical analysis, the Z-Scheme mechanism for the PEC degradation of TC·HCl over the TiO_2_ NTs/Ag/MoS_2_ meshy photoelectrode is proposed in Scheme 1. According to a previous report [22], the conduction band (CB) and valance band (VB) potentials of MoS_2_ are −0.12 eV and +1.78 eV, respectively. The CB and VB potentials of TiO_2_ are −0.29 eV and +2.91 eV, respectively. Under visible light irradiation, the photogenerated electrons (e^−^) of MoS_2_ are excited from its VB to CB, creating equal amount of holes (h^+^) on the valence band (R1). However, TiO_2_ cannot be excited by visible light due to its wide band gap (3.2 eV). The electrons from the extra circuit inject into the CB of TiO_2_ NTs, transfer to metallic Ag and then to the VB of MoS_2_, and finally combine with the holes located at VB of MoS_2_ (R2). In such a way, the electrons left in the CB of MoS_2_ with more negative potential value facilitate the formation of •O_2_^−^ (R3). The •O_2_^−^ radicals further participate in a side reaction to further generate •OH (R4,5,6). These active radical species of •O_2_^−^, •OH, and h^+^ have high oxidizability, which could take part in the PEC degradation of TC·HCl (R7). During these charge transfers, the metallic Ag could act as a cross-linking bridge for the formation of Z-scheme heterojunction, which facilitates the efficient separation of photogenerated electron-hole pairs and leads to a higher degradation efficiency.

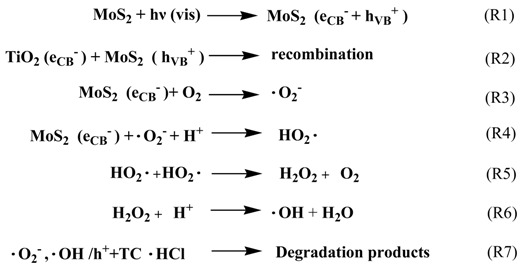


## 4. Conclusions

TiO_2_ NTs/Ag/MoS_2_ meshy photoelectrode was successfully prepared by decorating Ag nanoparticles and MoS_2_ nanosheets on the anodic oxidized TiO_2_ NTs/Ti mesh via photoassisted reduction and hydrothermal process. This composite showed remarkable photoelectrocatalytic activity and excellent stability for the PEC degradation of TC·HCl under visible light irradiation. The enhanced PEC activity can be attributed to the strong visible light adsorption, fast electron transfer velocity, and efficient separation of photogenerated electron-hole pairs. Since the TiO_2_ NTs/Ag/MoS_2_ mesh is solid and conductive, it can be used as electrode in photovoltaic devices, which opens up new prospects in environmental and energy applications, such as PEC degradation of pollutants, solar hydrogen evolution, and lithium ion batteries.

## Supplementary Materials

The following are available online at http://www.mdpi.com/2079-4991/8/9/666/s1, Figure S1: Element mapping pictures of TiO_2_ NTs/Ag/MoS_2_, Figure S2: Degradation efficiencies of TC·HCl under visible light irradiation: (a) with bias potential of 0.05 V, 0.1 V, 0.2 V, 0.3V, and 0.5 V; (b) direct photolysis, electrical catalysis, photocatalysis, and photoelectrocatalysis, Figure S3: XRD patterns of TiO_2_ NTs/Ag/MoS_2_: (a) before; (b) after PEC degradation of TC·HCl, Figure S4: ESR spectra of radical adducts trapped by DMPO in TiO_2_ NTs/Ag/MoS_2_ dispersions in dark and under visible light irradiation: (a) DMPO-•OH; (b) DMPO-•O_2_-.
