# Supplementary material for: TiO2 Nanotubes/Ag/MoS2 Meshy Photoelectrode with Excellent Photoelectrocatalytic Degradation Activity for Tetracycline Hydrochloride
Source: Nanomaterials (Basel). 2018 Aug 27;8(9):666. doi: 10.3390/nano8090666 (PMC6163688; doi:10.3390/nano8090666)
Supplement: Supplementary file 1 [file nanomaterials-08-00666-s001.pdf]

# $TiO_2$ nanotubes/Ag/$MoS_2$ meshy photoelectrode with excellent photoelectrocatalytic degradation activity for tetracycline hydrochloride

Tingting Li[1,2], Zhuhong Wang[1,2], Chaochao Liu[1,2], Chunmin Tang[1,2], Xinkai Wang[1,2], Gongsheng Ding[1,2],Yichun Ding[1,2], Lixia Yang[1,2,*]

[1]College of Environmental and Chemical Engineering, Nanchang Hangkong University, Nanchang 330063, China

[2]Key Laboratory of Jiangxi Province for Persisitent Pollutants Control and Resources Recyle, Nanchang Hangkong University, Nanchang 330063, China

## Supporting information

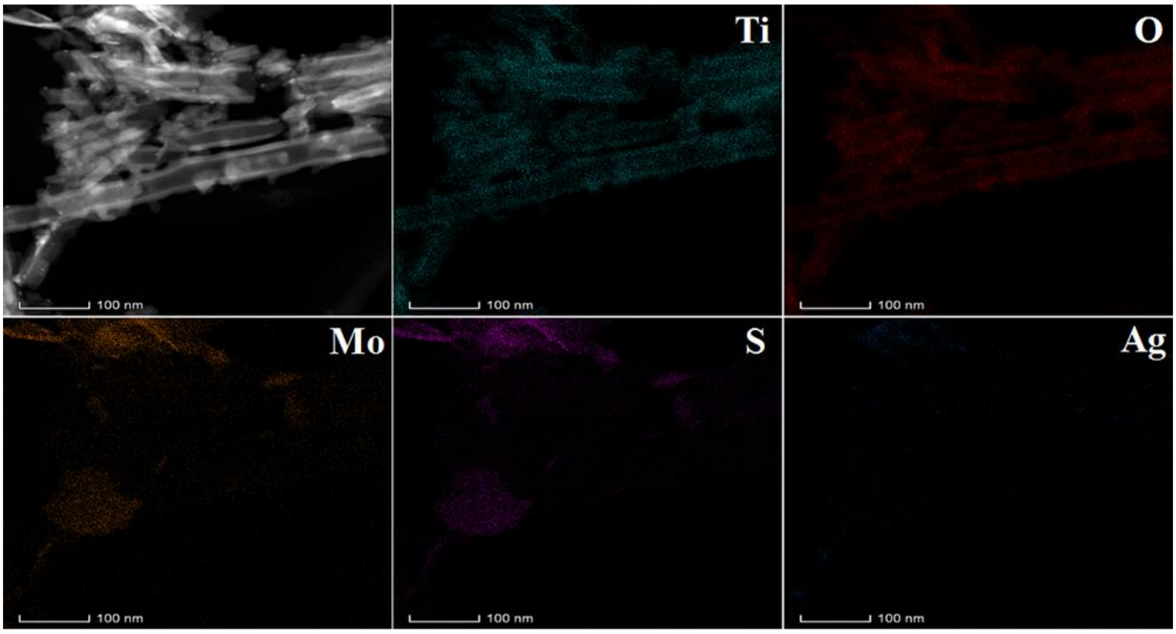


**Figure S1** Element mapping pictures of $TiO_2$ NTs/Ag/$MoS_2$.

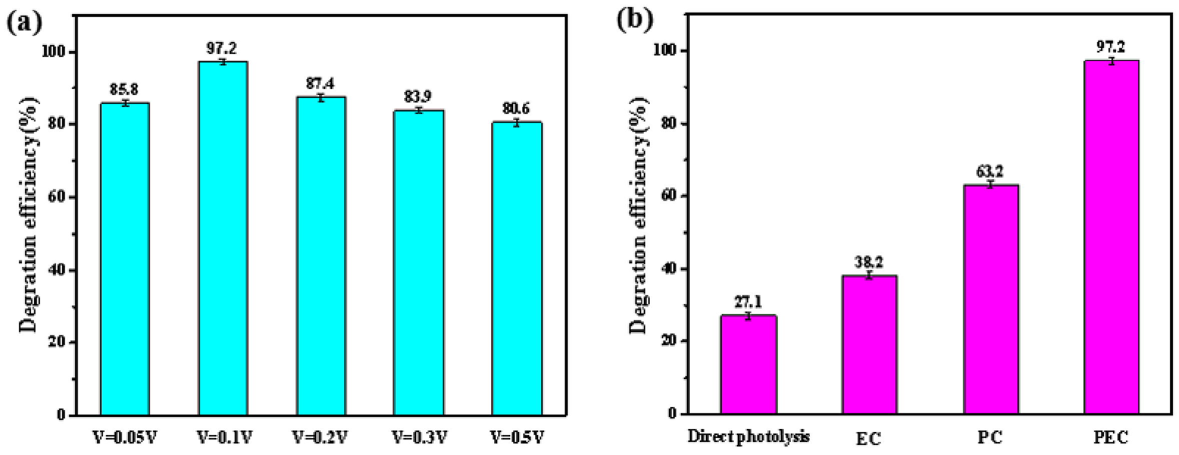


**Figure S2** Degradation efficiencies of TC HCl under visible light irradiation:(a)with bias potential of 0.05 V, 0.1 V, 0.2 V, 0.3V and 0.5 V; (b) direct photolysis, electrical catalysis, photocatalysis, photoelectrocatalysis.

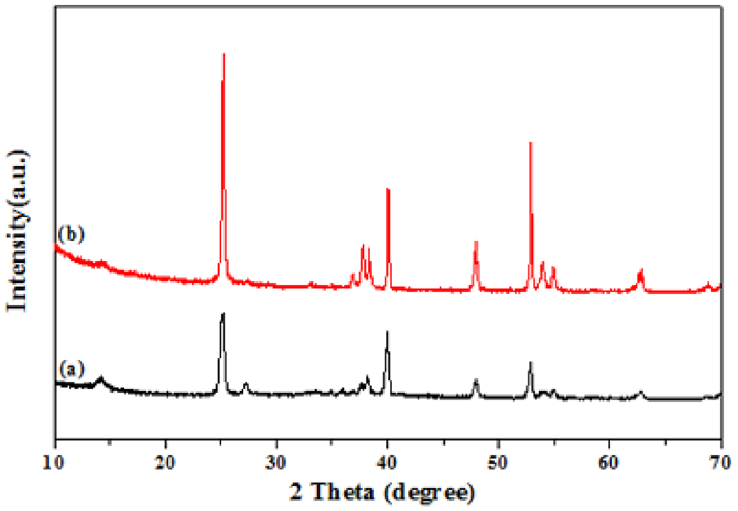


**Figure S3** XRD patterns of $TiO_2$ NTs/Ag/$MoS_2$: (a)before ;(b)after PEC degradation of TC HCl.

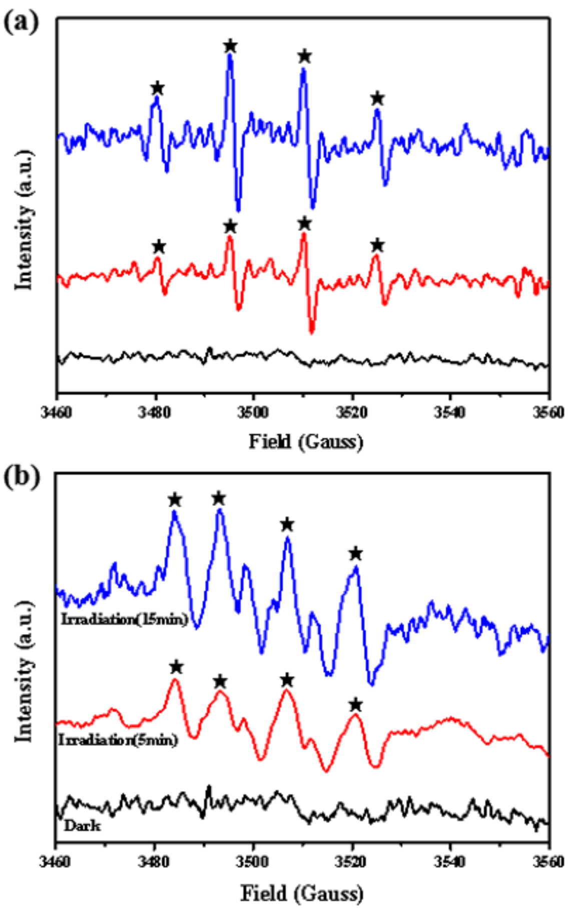


**Figure S4** ESR spectra of radical adducts trapped by DMPO in $TiO_2$ NTs/Ag/$MoS_2$ dispersions in dark and under visible light irradiation: (a)DMPO-•OH; (b)DMPO-•$O_2^-$.
